# Identification of hub genes predicting the development of prostate cancer from benign prostate hyperplasia and analyzing their clinical value in prostate cancer by bioinformatic analysis

**DOI:** 10.1007/s12672-022-00508-y

**Published:** 2022-06-30

**Authors:** Xi Chen, Junjie Ma, Chengdang Xu, Licheng Wang, Yicong Yao, Xinan Wang, Tong Zi, Cuidong Bian, Denglong Wu, Gang Wu

**Affiliations:** Department of Urology, Tongji Hospital, School of Medicine, Tongji University, 389 Xincun Road, Shanghai, 200065 China

**Keywords:** Benign prostate hyperplasia, Prostate cancer, Hub gene, Clinical value, Bioinformatic analysis

## Abstract

Prostate cancer (PCa) and benign prostate hyperplasia (BPH) are commonly encountered diseases in males. Studies showed that genetic factors are responsible for the occurrences of both diseases. However, the genetic association between them is still unclear. Gene Expression Omnibus (GEO) database can help determine the differentially expressed genes (DEGs) between BPH and PCa. Gene Ontology (GO) and Kyoto Encyclopedia of Genes and Genomes (KEGG) analysis were utilized to find pathways DEGs enriched. The STRING database can provide a protein–protein interaction (PPI) network, and find hub genes in PPI network. R software was used to analyze the clinical value of hub genes in PCa. Finally, the function of these hub genes was tested in different databases, clinical samples, and PCa cells. Fifteen up-regulated and forty-five down-regulated genes were found from GEO database. Seven hub genes were found in PPI network. The expression and clinical value of hub genes were analyzed by The Cancer Genome Atlas (TCGA) data. Except *CXCR4*, all hub genes expressed differently between tumor and normal samples. Exclude *CXCR4*, other hub genes have diagnostic value in predicting PCa and their mutations can cause PCa. The expression of *CSRP1*, *MYL9* and *SNAI2* changed in different tumor stage. *CSRP1* and *MYH11* could affect disease-free survival (DFS). Same results reflected in different databases. The expression and function of *MYC*, *MYL9*, and *SNAI2*, were validated in clinical samples and PCa cells. In conclusion, seven hub genes among sixty DEGs may be achievable targets for predicting which BPH patients may later develop PCa and they can influence the progression of PCa.

**Supplementary Information:**

The online version contains supplementary material available at 10.1007/s12672-022-00508-y.

## Introduction

Benign prostate hyperplasia (BPH) is common among elderly men over 70. Prostate cancer (PCa) is still have high incidence rates of all cancers and is a major reason to death in elderly men [1]. Although BPH and PCa are different diseases, in that BPH is a benign disease that arises in the transitional zone, and PCa is a malignant tumor that arises mainly in the peripheral zone, they are related. The relationship between BPH and PCa was first noted in the 1950s in studies of prostate glands. During the past 60 years, some studies have shown that BPH and PCa have some definite associations. Sommers first studied BPH and PCa on cadavers. In their work, BPH was found in 80% and 45% of cadavers with or without PCa, respectively [2]. Later, another study covered alike results [3]. In 2002, Hammarsten and Hogstedt reported that BPH which has a faster growing speed may be hazards for developing clinical PCa [4]. Another study proved that the volume of the prostate may be one of the reasons for the aggressiveness of PCa, and PCa located in small glands is more aggressive than that located in larger glands [5]. This means that BPH may affect the degree of malignancy of PCa. Orsted et al. followed 3,009,258 Danish men from 1980 to 2006. During 27 years of follow-up, they found that clinical BPH was linked with a raised risk of PCa and a higher risk of death by BPH [6].

In 2001, Luo et al. researched the genetic relationship between BPH and PCa, and found that 3215 genes were expressed differently between BPH and PCa samples [7]. Some studies have reported that gene expression could be a causal factor in the development of PCa from BPH and may even affect the degree of malignancy of PCa [8, 9]. Microarray technology and bioinformatic analysis have been extensively wielded to analyze differentially expressed genes (DEGs) and can be used to find functional pathways that will help us to better understand PCa [10]. Through gene expression profiling, some investigations have found many DEGs that play critical roles in the process of the development of PCa from BPH [8, 9]. However, the specific gene mutation of PCa from BPH and their clinical value in PCa are still unclear. So, the aim of the study is to find the potential gene markers which can predict the occurrence of PCa from BPH and then estimated the hub genes’ value in predicting PCa progression.

The Gene Expression Omnibus (GEO) database can provide many microarray datasets. Gene Ontology (GO), Kyoto Encyclopedia of Genes and Genomes (KEGG) pathway enrichment analysis and protein–protein interaction (PPI) network analyses have been performed to help us understand the potential mechanisms for the occurrence and progression of diseases. Thus, we used bioinformatic analysis to find key genes that may be important for development of PCa from BPH and further analyzed the clinical value of these genes in PCa. Then, based on genetic and clinical data from The Cancer Genome Atlas (TCGA) database, the diagnostic model was built to predict the hub genes of diagnostic value for PCa. Tumor staging and survival time were also used to analyze the role of hub genes in the progress of PCa. Logistic regression was used for predicting the mutation of hub genes leading to PCa. The expression of hub gene was validated in other databases. Next, we tested the expression three hub genes, *MYC*, *MYL9*, and *SNAI2*, in PCa based on clinical specimens. Finally, we chose a PCa cell line: C4-2, which can growth in an environment without androgen and express androgen receptor, to test the function of three hub genes in vitro. We found when transfected by MYC knock-down, MYL9 and SNAI2 overexpression plasmids, both cell invasion and proliferation decreased. The results indicated that these hub genes may be potential biomarkers for predicting the occurrence of PCa from BPH and they can also affect the progression of PCa.

## Materials and methods

### Microarray data

The GEO database (http://www.ncbi.nlm.nih.gov/geo/) at the National Center for Biotechnology Information (NCBI) is a communal database that provides a genomics data repository of gene expression, chip, and microarray data [11]. The criteria for GSE data included in the study as follow: (1) The GSE samples have complete gene expression data from high-throughput sequencing and can be downloaded from GEO database. (2) The GSE samples data included both BPH samples and PCa samples. (3) There is a clear definition of BPH and PCa samples. Then we found three datasets GSE5377, GSE104749 and GSE30994 met our criteria. Then we downloaded the three datasets from GEO database. GSE5377 included 3 BPH samples and 17 PCa samples. GSE104749 included 4 BPH samples and 4 PCa samples. GSE30994 included 3 BPH samples and 3 PCa samples. Overall, 10 BPH and 24 PCa samples were enrolled in our study.

### Data handling and DEGs searching

The primary data were got and normalized by R software (version 4.0.2). According to comments of the documents, the expression matrix including probe ID was substituted by the corresponding gene ID, and if there were multiple probes that corresponded to the same gene, the average value was calculated using the R software by bioconductor package (version 4.0) for further study [12]. Then all genes of each data set were searched using the limma R package (version 4.0), and genes with an adjusted *P*-value < 0.05 and |log2fold change (FC)| > 1 were considered DEGs. Then, we used the online web tool, Venn diagrams (http://bioinformatics.psb.ugent.be/webtools/Venn/) to find the integrated DEGs. In addition, the up-regulated and down-regulated genes were downloaded for further study.

### GO and KEGG pathway analysis of DEGs

The Database for Annotation, Visualization, and Integrated Discovery (DAVID) v6.8 (https://david.ncifcrf.gov/) was used to perform GO functional and KEGG pathway analyses of the integrated DEGs [13]. The GO functional analysis of integrated DEGs involved three parts: biological processes (BP), cell components (CC), and molecular functions (MF). *P* < 0.05 was considered statistically statistical differences [14].

### PPI network and module analysis

A PPI network of the integrated DEGs was structured by online tool the Search Tool for the Retrieval of Interacting Genes (STRING) database with the default medium confidence (0.4) (http://www.string-db.org/) [15]. It helped us to find the key genes and critical gene modules participated in the promotion of BPH to PCa. Cytoscape software was used for reconstructing the PPI network, and module and GO analyses were carried out by two plug-ins in Cytoscape, Molecular Complex Detection (MCODE) and Biological Network Gene Ontology tool (BiNGO), to clarify the biological significance of gene modules from BPH to PCa. *P* < 0.05 indicated a significant difference, and these genes were designated as hub genes [16].

### Construction of risk prediction model and survival analysis

Hub gene expression between normal prostate specimens and PCa tissues was compared using gene expression profiling interactive analysis (GEPIA; http://www.gepia.cancer-pku.cn/) dependent on TCGA database (including 492 tumor samples and 152 normal samples) [17]. Logistic regression was performed to screen the hazard ratios of hub genes changes leading to PCa. A nomogram was built to predict the risk value of the hub genes. A forest map was utilized to show the hazard ratios more intuitively. Moreover, the prognostic value of gene was enucleated by GEPIA. Then, overall survival (OS), disease-free survival (DFS) was analyzed too.

### Construction of the diagnostic model and decision curve analysis

To further analyze the hub genes’ diagnostic value for PCa, we collected the gene expression of hub genes and clinical data from TCGA databases (https://portal.gdc.com) [18]. GraphPad Prism 7 (GraphPad Software, Inc., San Diego, CA) was used to draw the receiver operating characteristic (ROC) curve and decision curve analysis (DCA) was carried out by R software Risk Model Decision Analysis (rmda) package (version 4.0).

### Expression of hub genes at different tumor stages

Tumor-Node-Metastasis (TNM) classification of malignant tumors is commonly used to assess the tumor severity [19]. Hub gene expression in different TNM stage of PCa was analyzed on TCGA data.

### Validation of hub gene expression based on Chinese PCa patients and different databases

We downloaded the RNA-sequence data of Chinese PCa patients from Chinese Prostate Cancer Genome and Epigenome Atlas (CPGEA; http://www.cpgea.com/) and tested hub genes expression in Chinese PCa patients (136 tumor tissues and 136 normal tissues). We further analyzed the hub genes’ expression in normal prostate samples and PCa specimens on the UALCAN database (http://ualcan.path.uab.edu/) and The Human Protein Atlas (https://www.proteinatlas.org/) [20, 21].

### Clinical specimen collection

The PCa patients participated in the study was confirmed had BPH before. The methods used for collecting the samples were approved by the Ethics Committee of Tongji Hospital, School of Medicine, Tongji University (SBKT-2021-220). Patients who provided the samples were familiar with the process of the experiment and gave informed consent.

### Cell culture and transfection

C4-2 PCa cells were purchased from the Chinese Academy of Science Cell Bank (Shanghai, China). C4-2 cells were cultured in Roswell Park Memorial Institute (RPMI) 1640 medium (Sigma, Darmstadt, Germany, Catalog No. R8758) with 10% fetal bovine serum (FBS) (Gibco, Thermo Fisher Scientific, Waltham, MA, USA, Catalog No. 10091). C4-2 cells were transfected with MYC breakdown (shMYC) plasmids, MYL9 overexpression (oeMYL9) plasmids, and SNAI2 overexpression (oeSNAI2) plasmids (constructed by fenghbio company Hunan, China) by Lipofectamine 2000 (Thermo Fisher Scientific, Catalog No. 11668019) according to the manufacturers’ instructions. The shMYC plasmid sequence is: CCTGAGACAGATCAGCAACAA.

### Antibodies

Rabbit monoclonal antibodies against c-MYC (Catalog No. ab32072) and MYL-9 (Catalog No. ab191312) was purchased from abcam company (Cambridge, UK). Mouse monoclonal antibodies against SNAI2 (Catalog No. ab51772) and anti-GAPDH (Catalog No. ab8245) was purchased from abcam company, too. HRP AffiniPure Goat Anti-Rabbit IgG (Catalog No. A0216) and HRP AffiniPure Goat Anti-Mouse IgG (Catalog No. A0208) were purchased from Beyotime Biotechnology Company (Shanghai, China).

### RNA extraction and qRT-PCR

The total RNA was extracted from tumor and para-cancerous tissues of patients and cells utilizing TRIzol Reagent (Sigma-Aldrich, St. Louis, MO, USA, Catalog No. T9424). cDNA was transcribed using the reverse transcription kit (Advantage® RT-for-PCR Kit, Takara Bio Inc., Kusatsu, Japan, Catalog No. 639505). Finally, we measured the volume of cDNA using real-time PCR reagents and a kit (TB Green® Premix Ex Taq™ II, Takara Bio Inc., Catalog No. RR420A) according to the manufacturer’s descriptions. The following primers of c-MYC, MYL9, SNAI2 and GAPDH were shown in Table 1. The 2^−ΔΔCt^ method was used to quantify mRNA expression levels.

**Table 1:** Primers used for the qRT-PCR

Gene name	Primer sequence
c-MYC	Forward: 5′-AGCGACTCTGAGGAGGAACAA-3′
	Reverse: 5′-TGGGCTGTGAGGAGGTTTG-3
MYL9	Forward: 5′-AACATGTCCAGCAAACGTGC-3′
	Reverse: 5′-GCGAAGACATTGGAGGTGG-3′
SNAI2	Forward: 5′-GGACTAGTATGCCGCGCTCCTTCCTGGTC-3′
	Reverse:5′-CGGAATTCTCAGTGTGCTACACAGCAGCCAGATTC-3′
GAPDH	Forward: 5-GGAGCGAGATCCCTCCAAAAT-3′
	Reverse: 5′-GGCTGTTGTCATACTTCTCATGG-3′

### Western blot

Protein was extracted with RIPA lysis buffer from tissues and cells. Protein samples were treated with Dual Color Protein Loading Buffer [Thermo Fisher Scientific, Waltham, MA, USA, Catalog No. NP0007). SDS-PAGE gels (10% and 15%) were used to separate proteins, followed by transfer to nitrocellulose membranes (Merck KGaA, Darmstadt, Germany, Catalog No. 71078)]. Protein-Free Rapid Blocking Buffer (Thermo Fisher Scientific, Catalog No. 37584) was utilized to block the membranes. Then the membranes were incubated overnight at 4 °C with primary antibodies against c-MYC (1:1000), MYL9 (1:1000), SNAI2 (1:1000) and GAPDH (1:1000). The next day, 1xTBST was used to wash the membranes three times (10 min. each). Then, the membranes were incubated at room temperature for 1 h with a matched secondary antibody (1:1000). Lastly, the membranes were exposed to X-ray film (FluorChem R, Protein Sample, California, USA).

### Immunohistochemistry (IHC)

The expression of MYC, MYL9, and SNAI2 in clinical patients’ specimens was detected by IHC. Tumor samples were fixed by formalin and embedded into paraffin. Four-micrometer thick sections were cut from the samples and fixed. Sections were antigen retrieved and immunostaining was performed as described [22]. Anti-MYC antibody (1:1000), anti-MYL9 antibody (1:400) and anti-SNAI2 antibody (1:500). Two experienced pathologists (unaware of tissue information) independently evaluated and scored the intensity of IHC.

### Cell invasion assay

After 48 h of transfection, approximately 1 × 10^5^ C4-2 cells and 150 µL 2% fetal bovine serum FBS + 1640 culture medium was put in the upper chamber, and 10% FBS + 1640 culture medium was placed in the lower cubicle. After 48 h, cells were fixed with 4% paraformaldehyde fixative solution. The cells were stained with crystal violet and observed by an Olympus microscope (Olympus Corp. Tokyo, Japan). ImageJ was utilized to count cell numbers.

### Cell proliferation assay

After 48 h of transfection, about 1000 C4-2 cells were placed in each well of a 96-well plate. Each set was repeated three times. The proliferation of cells in 0, 24, 48, and 72 h were detected by Cell Counting Kit-8 (CCK-8) (Solarbio, Beijing, China, Catalog No. CA1210). The optical density (OD) at 450 nm was measured by enzyme labeling (LD942, Beijing, China).

### Statistical analysis

The matrix data was handled with R version 4.0.2 (Institute for Statistics and Mathematics, Vienna, Austria; https://www.r-project.org). For descriptive statistics, mean ± standard deviation was used for continuous variables with normal distributions, whereas the median (range) was used for continuous variables with abnormal distributions. Categorical variables were described by counts and percentages. Hazard ratios (HRs), the 95% confidence interval (95% CI), and P values were used as statistical metrics. Two-tailed *P* < 0.05 was deemed as statistically significant.

## Results

### Identification of DEGs

**Fig. 1:**
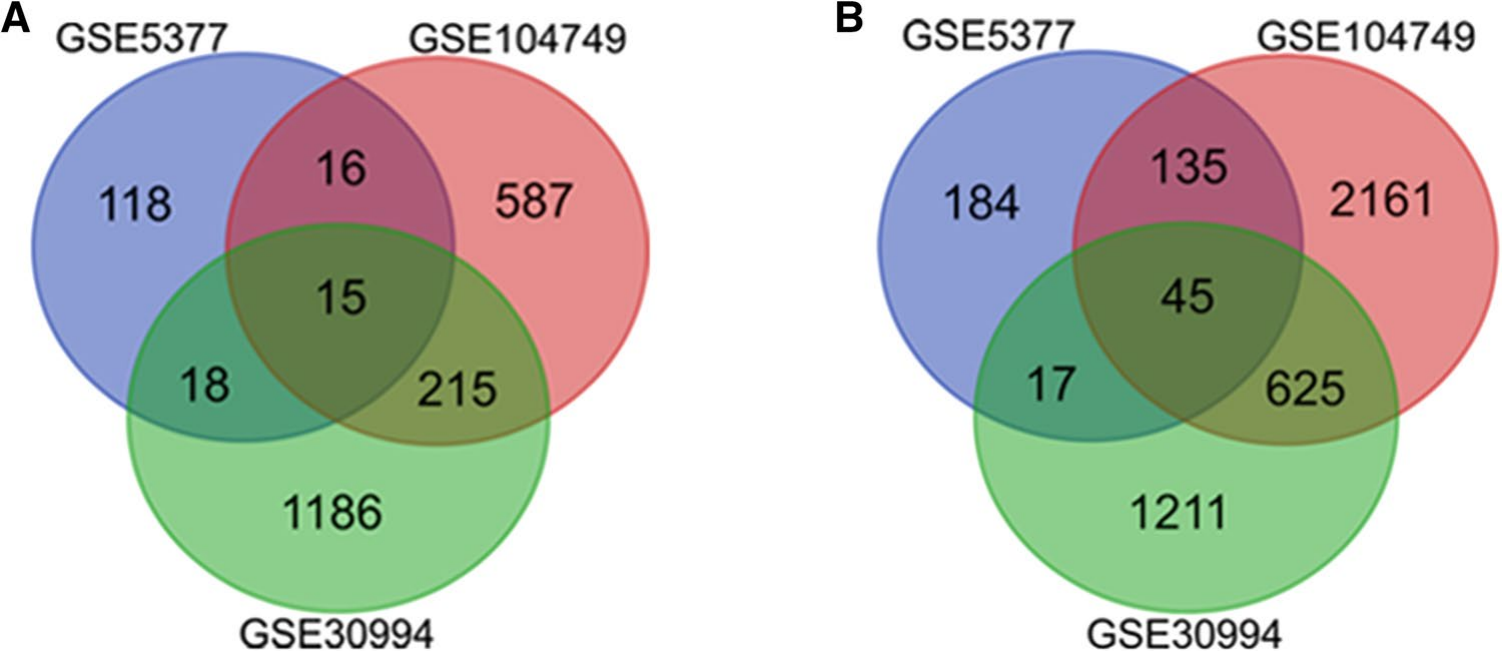
Venn map reflects DEGs between BPH and PCa from GSE5377, GSE104749 and GSE30994. **A** The up-regulated DEGs from GSE5377, GSE104749 and GSE30994. **B** The down-regulated DEGs from GSE5377, GSE104749 and GSE30994

Gene expression datasets GSE5377, GSE107479, and GSE30994 were acquired from GEO database. The GSE5377 dataset included 547 DEGs, with 167 up-regulated genes and 380 down-regulated genes. The GSE104749 dataset included 3790 DEGs consisting of 833 up-regulated genes and 2957 down-regulated genes. The GSE30994 dataset contained 3790 DEGs, including 1429 up-regulated genes and 1872 down-regulated genes. The up and down regulated DEGs was shown in Venn map (Fig. 1). In total, 15 up-regulated DEGs and 45 down-regulated DEGs were included. The characteristic of sixty DEGs was shown in Table S1.

### Hub gene finding in PPI network

**Fig. 2:**
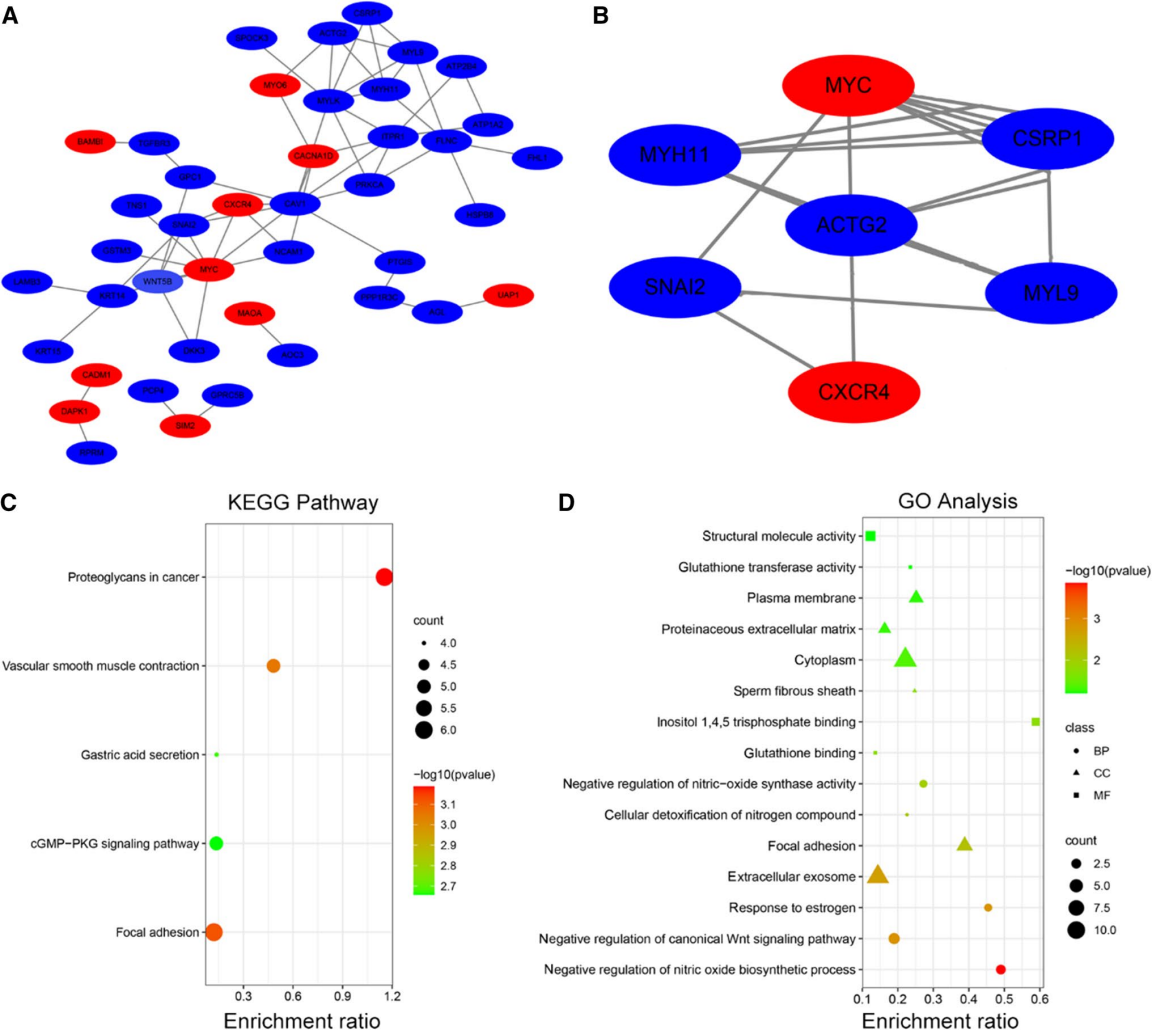
PPI network, GO, and KEGG pathways were shown. **A** The PPI network of all DEGs. **B** The interaction of 7 hub genes in PPI network. **C** KEGG pathways of DEGs were shown in scatter map. **D** GO analysis of DEGs was exhibited in scatter map. Up-regulated gene reflects in Red and down-regulated gene reflects in Blue

The PPI network of DEGs was constructed to help find the hub genes by SRTING. The most significant module was obtained using, MCODE and BiNGO, two plug-in Cytoscape software with *P* < 0.05, and 7 hub genes, *MYC*, *CXCR4*, *CSRP1*, *SNAI2*, *MYL9*, *ACTG2*, and *MYH11* were found (Fig. 2A). Moreover, the contact of the 7 hub genes was also analyzed (Fig. 2B).

### GO and KEGG pathway analysis of DEGs

Try to find the pathways which DEGs enriched, we used DAVID to find the pathways. In the KEGG pathway analysis, we found that the genes were mainly enriched in proteoglycans in cancer (Fig. 2C). In GO analysis, we found that the genes mainly enriched in inositol 1, 4, 5 trisphosphate binding and negative regulation of nitric oxide biosynthetic process (Fig. 2D).

### Expression of hub genes in TCGA database

**Fig. 3:**
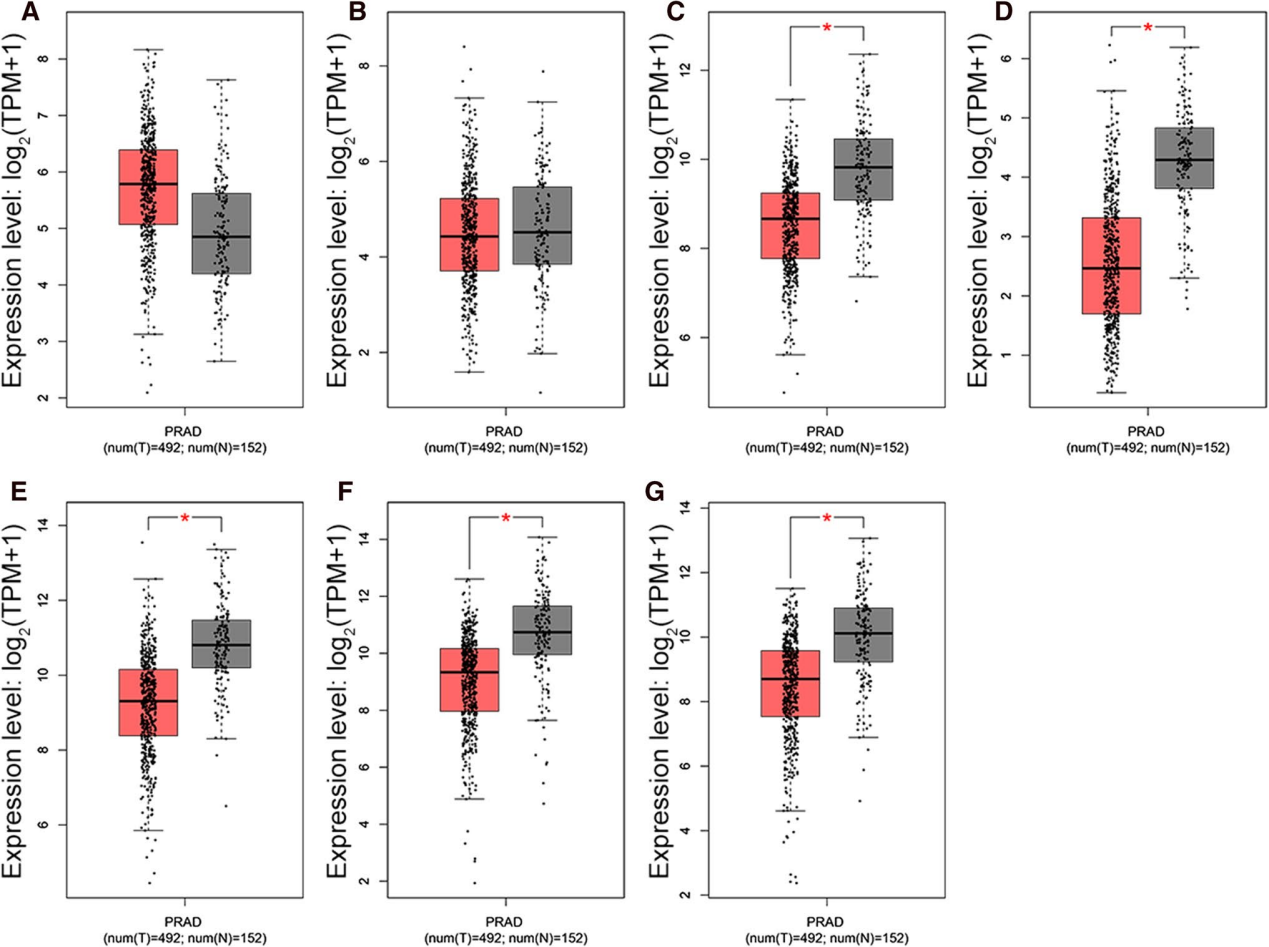
The expression of hub genes between normal prostate and PCa samples from GEPIA depend on TCGA and GTEx data. **A** *MYC*, **B** *CXCR4*, **C** *CSRP1*, **D** *SNAI2*, **E** *MYL9*, **F** *ACTG*2, **G** *MYH11*. Red color represents tumor samples and gray color represents normal samples. * represents statistical differences between two groups (*P* < 0.05)

To further confirm that the hub genes could be important factors leading to PCa, we used the GEPIA database to compare the hub genes’ expression in normal and PCa specimens. We found the hub genes were expressed differently in normal samples and PCa specimens, except for *MYC* and *CXCR4* (Fig. 3).

### ROC curve and decision curve analyses

**Fig. 4:**
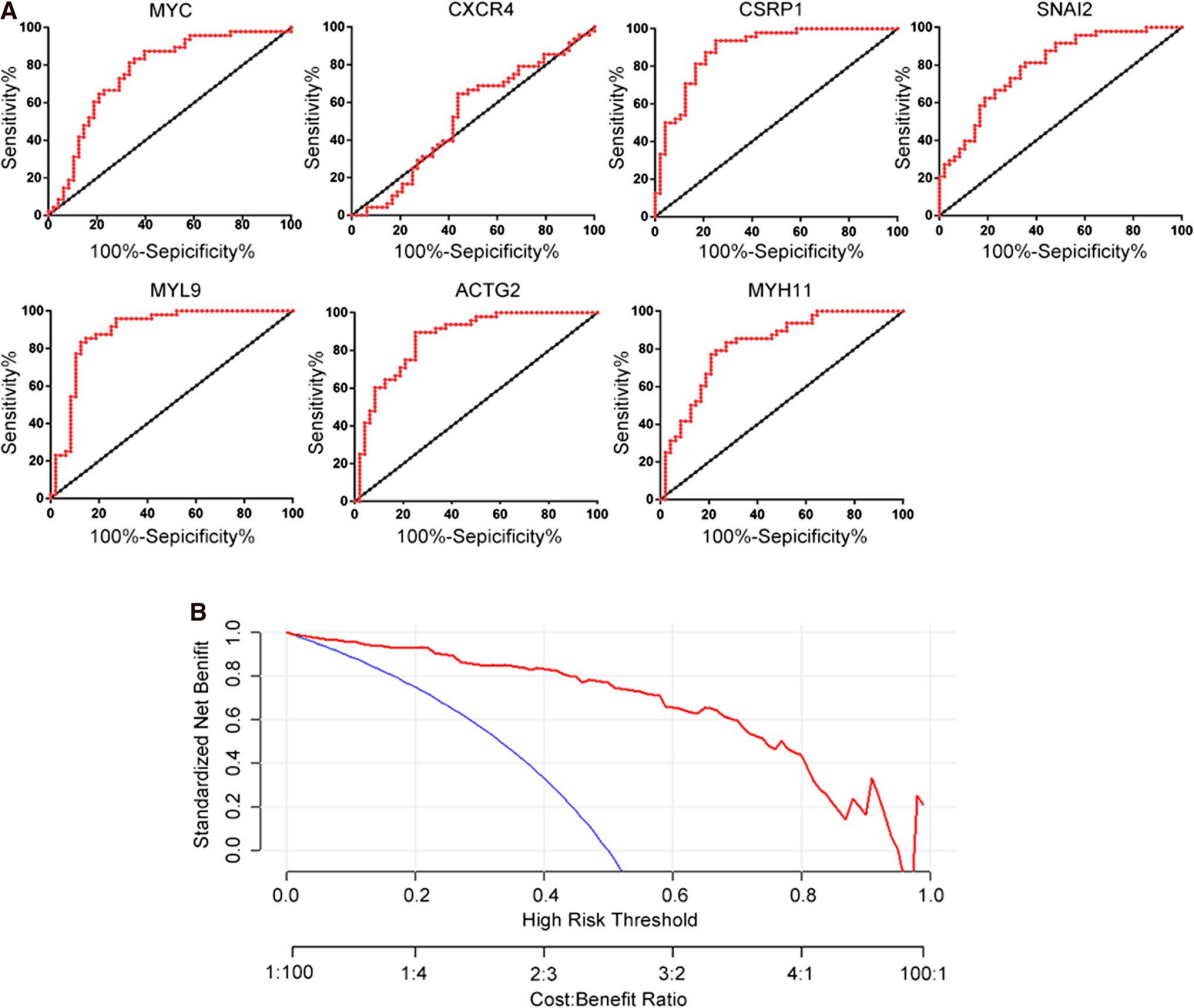
The diagnostic model of hub genes was constructed. **A** The ROC curve of hub genes was constructed in predicting PCa. **B** The DCA of 7 hub genes was draw in predicting PCa

The ROC curve was used to evaluate the hub genes’ ability to diagnose PCa. We found that *CXCR4* had poor diagnostic efficacy, with an Area Under Curve (AUC) of 0.5198 (95% CI 0.4316–0.6079; *P* = 0.6419). The other hub genes had perfect diagnostic values (*P* < 0.05). *MYC* had an AUC of 0.7553 (95% CI 0.6773–0.8332), *CSRP1* had an AUC of 0.8764 (95% CI 0.8294–0.9234), *SNAI2* had an AUC of 0.7399 (95% CI 0.7830–0.8968), *MYL9* had an AUC of 0.8300 (95% CI 0.7727–0.8873), *ACTG2* had an AUC of 0.8499 (95% CI 0.7956–0.9042), and *MYH11* had an AUC of 0.8651 (95% CI 0.8103–0.9198) (Fig. 4A and Table 2). This means that the other hub genes had significant diagnostic values. In addition, we made DCA to value the total value of these hub genes in predicting PCa (Fig. 4B).

**Table 2:** The diagnostic value of hub genes in PCa

Id	P value	AUC and 95% CI
MYC	0.0027	0.7553 (0.6773–0.8332)
CXCR4	0.6419	0.5198 (0.4316–0.6079)
CSRP1	0.0009	0.8764 (0.8294–0.9234)
SNAI2	0.0079	0.7399 (0.7830–0.8968)
MYL9	0.0153	0.8300 (0.7727–0.8873)
ACTG2	0.0178	0.8499 (0.7956–0.9042)
MYH11	1.76E-07	0.8651 (0.8103–0.9198)

### Hub gene expression at different tumor stages

**Fig. 5:**
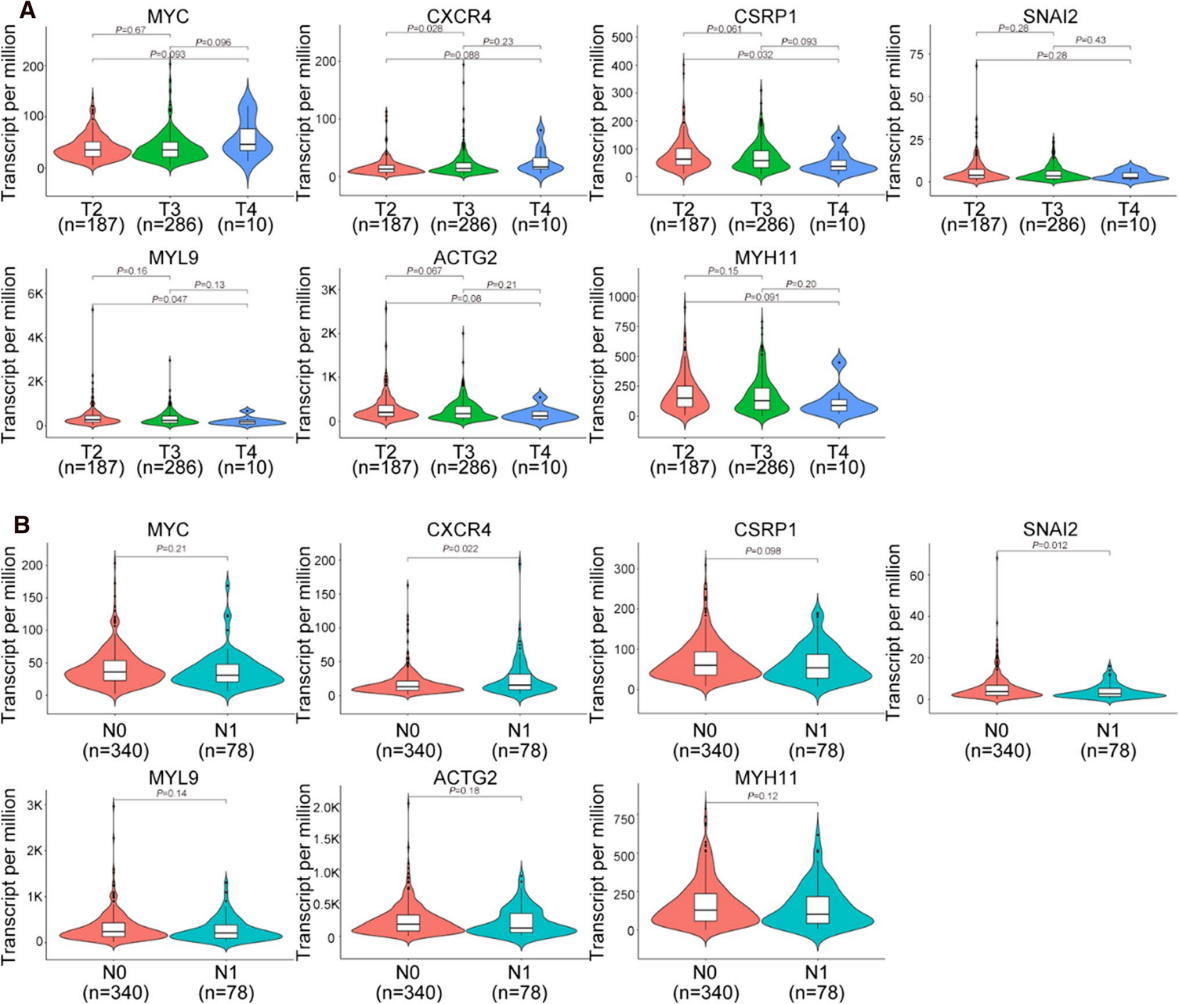
The expression of hub genes in different tumor stages depend on TCGA data. **A** The expression of hub genes in different Tumor (T) stage depend on TNM classification of malignant tumors. **B** The expression of hub genes in different Node (N) stage depend on TNM classification of malignant tumors

Further, we tried to find whether these hub genes can affect PCa progression. Using the clinical data downloaded from TCGA database, we then analyzed hub genes’ expression at different tumor stages. We analyzed the hub genes’ expression in different TNM classification of malignant tumors. We found that some hub genes expression will also change when PCa progression. For example, the expression of *CXCR4* will increase significantly from T2 tumor stage to T3 tumor stage (*P* = 0.028). *CSRP1* and *MYL9* will decrease when tumor progressed from T2 to T4 (*P* = 0.032 and *P* = 0.047) (Fig. 5A). The expression level of *CXCR4* and *SNAI2* will change when node metastasis happened (*P* = 0.022 and *P* = 0.012) (Fig. 5B).

### Risk prediction model and survival analysis

**Fig. 6:**
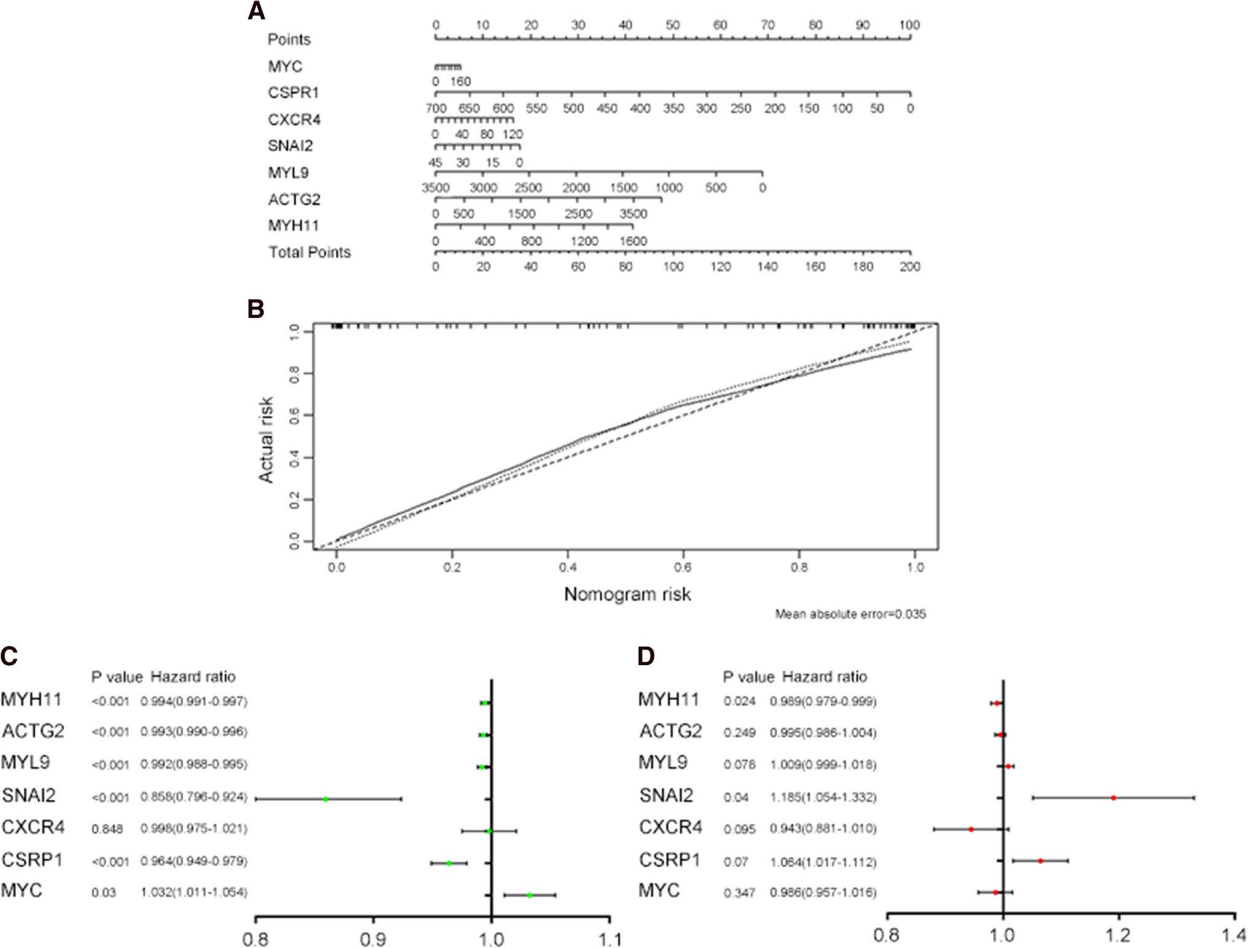
Risk prediction model of hub genes mutation in leading to PCa on TCGA data. **A** The nomogram of hub genes expression changes in causing PCa. **B** Calibration plot of actual risk probability and nomogram risk of nomogram. **C** The forest map of single factor of regression analysis of hub genes in causing PCa. **D** The forest map of multiple factors of regression analysis of hub genes in leading to PCa

**Fig. 7:**
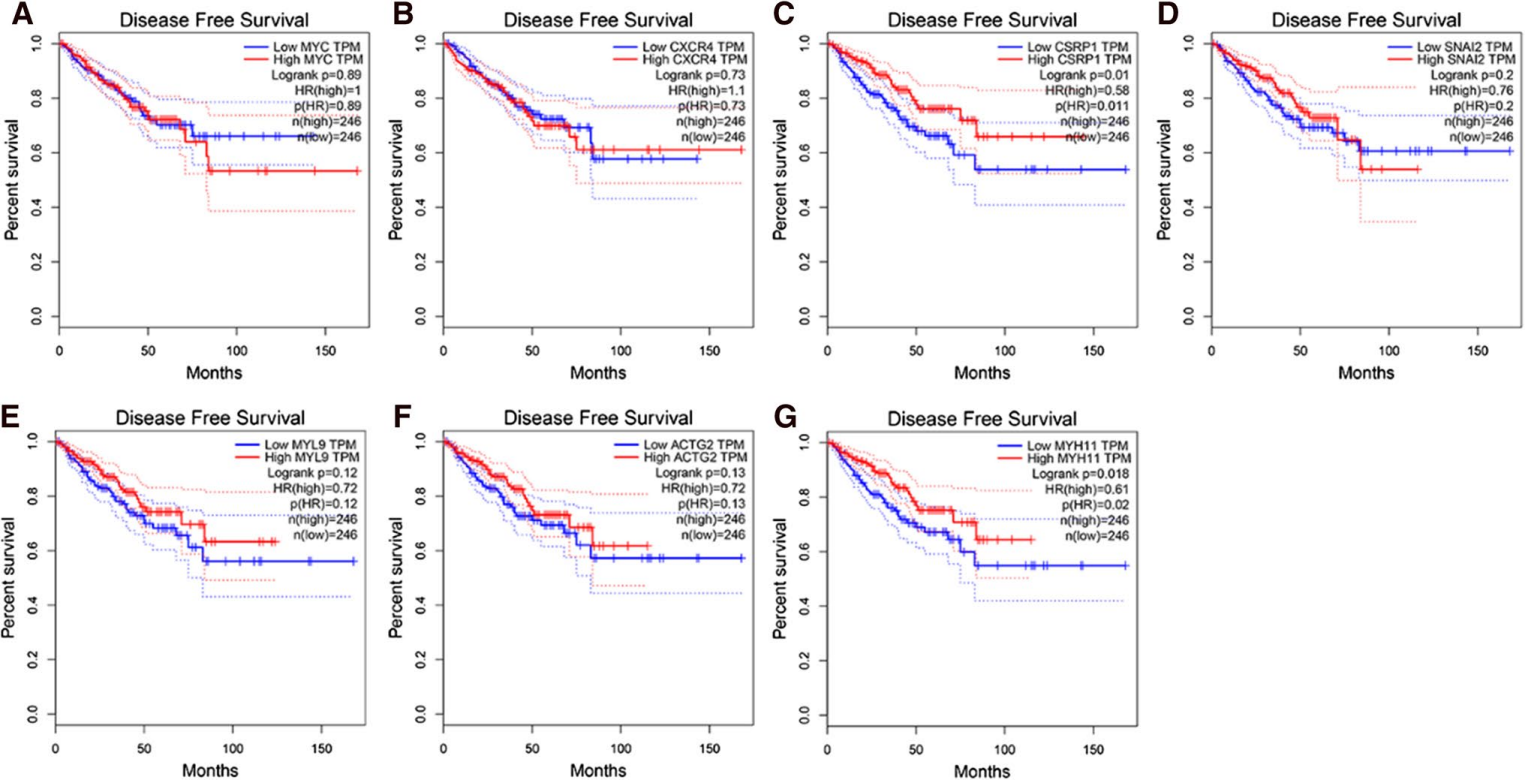
Survival analysis of hub genes effect in PCa patients’ DFS on TCGA data. **A** *MYC*, **B** *CXCR4*, **C** *CSRP1*, **D** *SNAI2*, **E** *MYL9*, **F** *ACTG2*, **G** *MYH11*

Logistic regression was predictive analysis model in predicting disease progress. Here, we made logistic analysis to predict hub genes’ expression in resulting PCa. The nomogram was constructed to forecast the probability of hub gene mutation leading to PCa (Fig. 6A). A calibration curve was made to verify the nomogram (Fig. 6B). Single factor and multi-factor regression showed the hub genes’ mutation risk in causing PCa (Fig. 6C, D). In single factor logistic regression forest map, we found that except *CXCR4*, (*P* = 0.848) all other hub genes may be risk factors in the occurrence of PCa (Fig. 6C). However, in the multiple factor logistic regression forest map, we found that only *SNAI2* (*P* = 0.04) and *MYH11* (*P* = 0.024) may be risk factors in leading to PCa (Fig. 6D). The residuals plot and the normal P–P plot of standardized residuals of logistic regression were used to test the effect of the regression model (Figure S1A, B). We further found that the hub gene *CSRP1* and *MYH11* can affect patients’ DFS (Fig. 7). However, no hub genes had an effect on OS (Figure S2).

### Hub gene expression in Chinese PCa patients and different databases

**Fig. 8:**
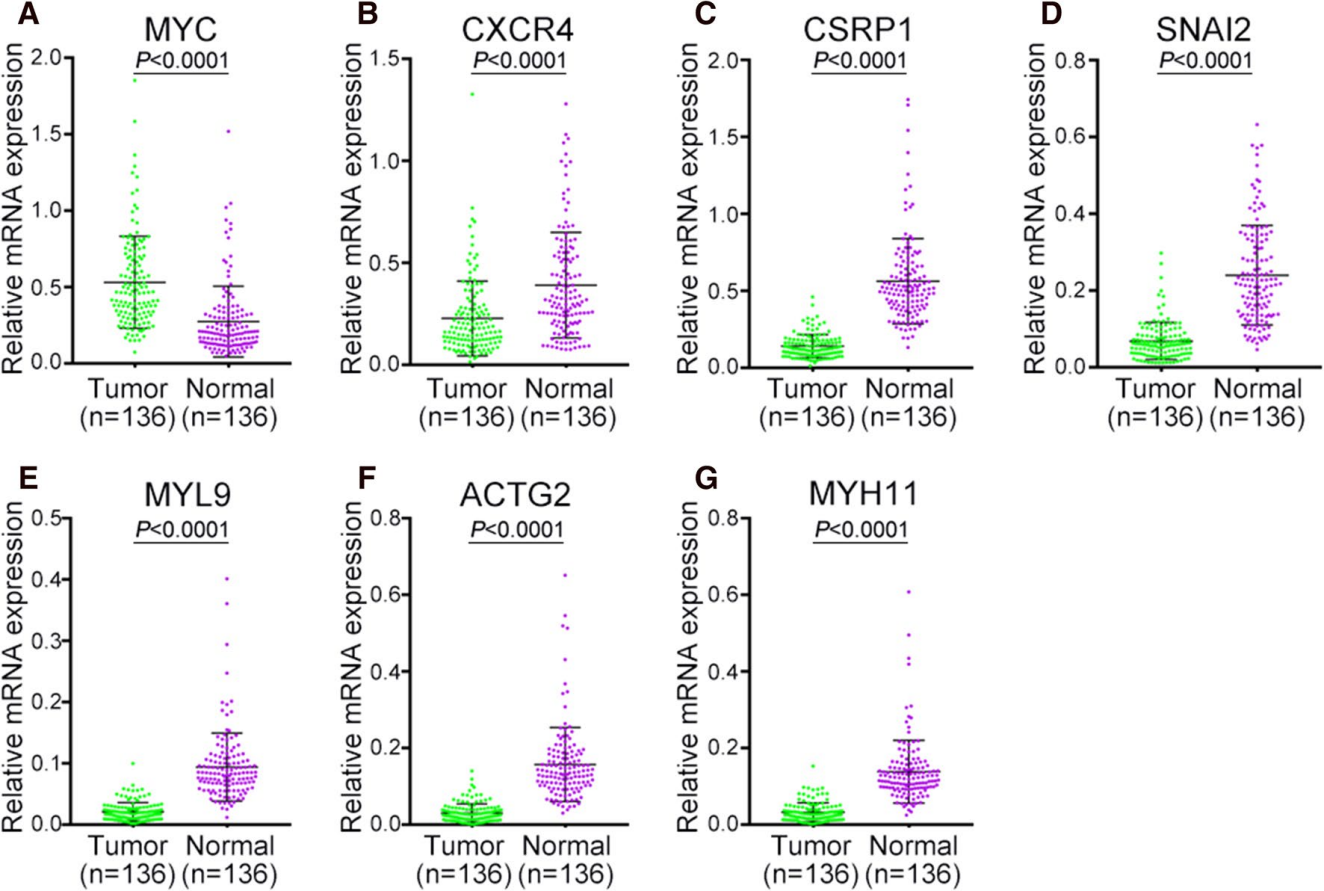
The expression of hub gene in Chinese PCa patients. **A** *MYC*, **B** *CXCR4*, **C** *CSRP1*, **D** *SNAI2*, **E** *MYL9*, **F** *ACTG2*, **G** *MYH11*

As the data from TCGA mainly included western people’s data, we tried to analyze the hub genes’ expression in Chinese PCa patients. We downloaded the RNA-sequence data of Chinese patients from CPGEA database. We found all these hub genes expressed differently in Chinese patients including *CXCR4* (Fig. 8). Further, we used the UALCAN and The Human Protein Atlas databases to compare the hub genes’ expression in normal and tumor specimens. Like the results found above, the other 6 hub genes except *CXCR4* were expressed differently in normal prostate tissue and PCa samples (Figure S3).

### Validation in clinical specimens

**Fig. 9:**
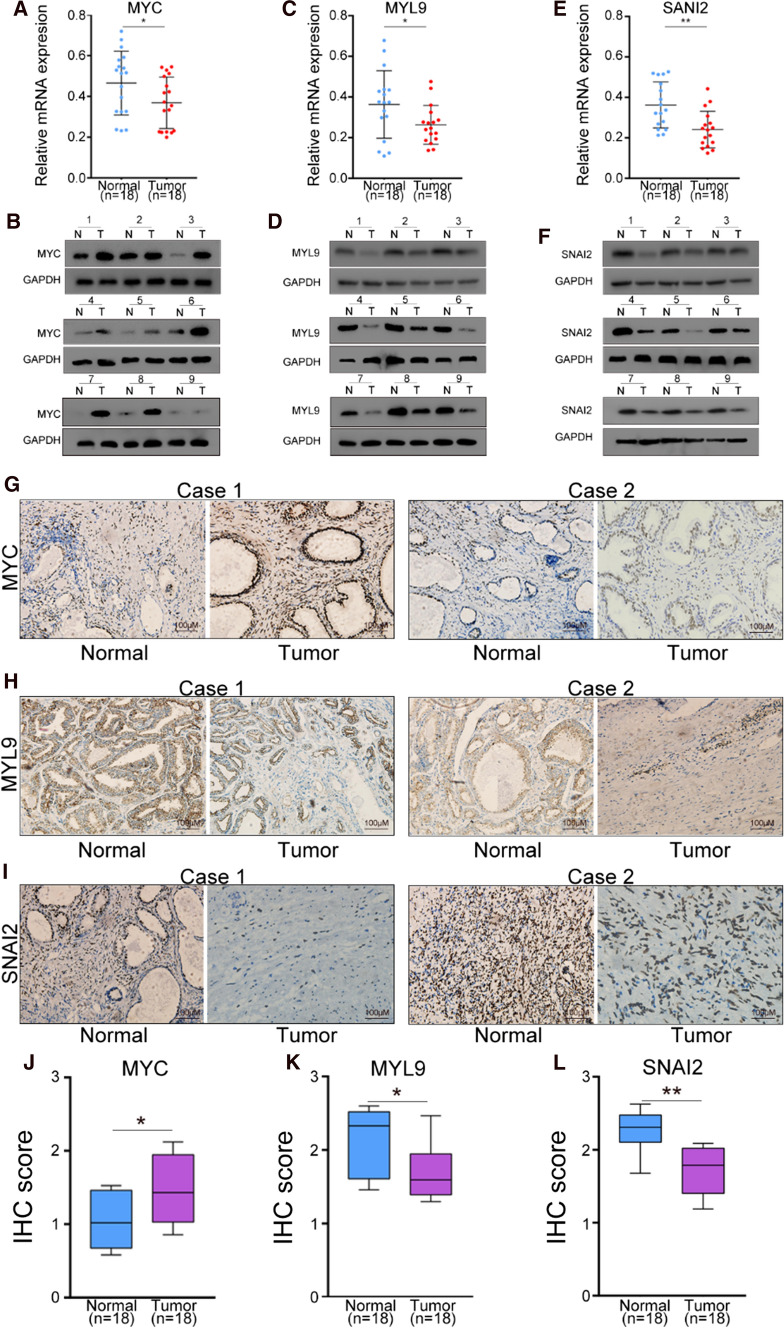
The expression of MYC, MYL9 and SNAI2 in patients with PCa from BPH in clinical specimens. **A**, **B** The mRNA and protein expression level of MYC between cancer tissues and para-cancerous normal tissues. **C**, **D** The expression of MYL9 at mRNA and protein level between cancer tissues and para-cancerous normal tissues. **E**, **F** The SNAI2 mRNA and protein expression of patients from both tumor tissues and para-cancerous normal tissues. **G**–**I** The Immunohistochemical results of MYC, MYL9 and SNAI2 in patients’ specimens from both cancer tissues and para-cancerous normal tissues. **J**–**L** The IHC score reflect the expression level of MYC, MYL9, and SANI2 in PCa specimens. The expression level of mRNA and protein was used GAPDH as inner control. * represents *P* < 0.05, ** represent *P* < 0.01, *** represent *P* < 0.001. N represents normal tissues, and T represents tumor tissues

Then, we tried to ensure whether these hub genes’ expression will change when PCa occurred from BPH. We collected clinical specimens from PCa patients. The PCa patients included in the study were confirmed to have BPH before. The clinical indexes of patients included in the study was shown in Table support 2 (Table S2). *MYC* and *CXCR4* was up-regulated genes. However, *CXCR4* expressed no difference in GEPIA and UALCAN. So, we choose *MYC,* as the upregulation gene, for experiment validation. In addition, there are 5 hub genes down-regulated. All the down-regulated genes expressed differently in TCGA. In ROC curve analysis, we found that *MYL9* and *SNAI2* has the lowest AUC among 5 down-regulated hub genes. That means the two hub genes may have minimum authenticity among 5 down-regulated hub genes in predicting PCa. So, we chose *MYL9* and *SNAI2* as the down-regulated genes for further study. We included 18 patients who were diagnosed with PCa with previous diagnoses of BPH in the study. We found that *MYC* was truly increased in both mRNA and protein level when PCa happened (Fig. 9A, B). *MYL9* expressed lower in tumor tissues than normal tissues at mRNA and protein level (Fig. 9C, D). Meanwhile, *SNAI2* was also down-regulated in PCa sample tissues than para-cancerous normal tissues. (Fig. 9E, F) At the same time, the IHC results reflect the same trend with the results as qRT-PCR and western blot (Fig. 9G–L).

### Hub genes influence on C4-2 PCa cell line

**Fig. 10:**
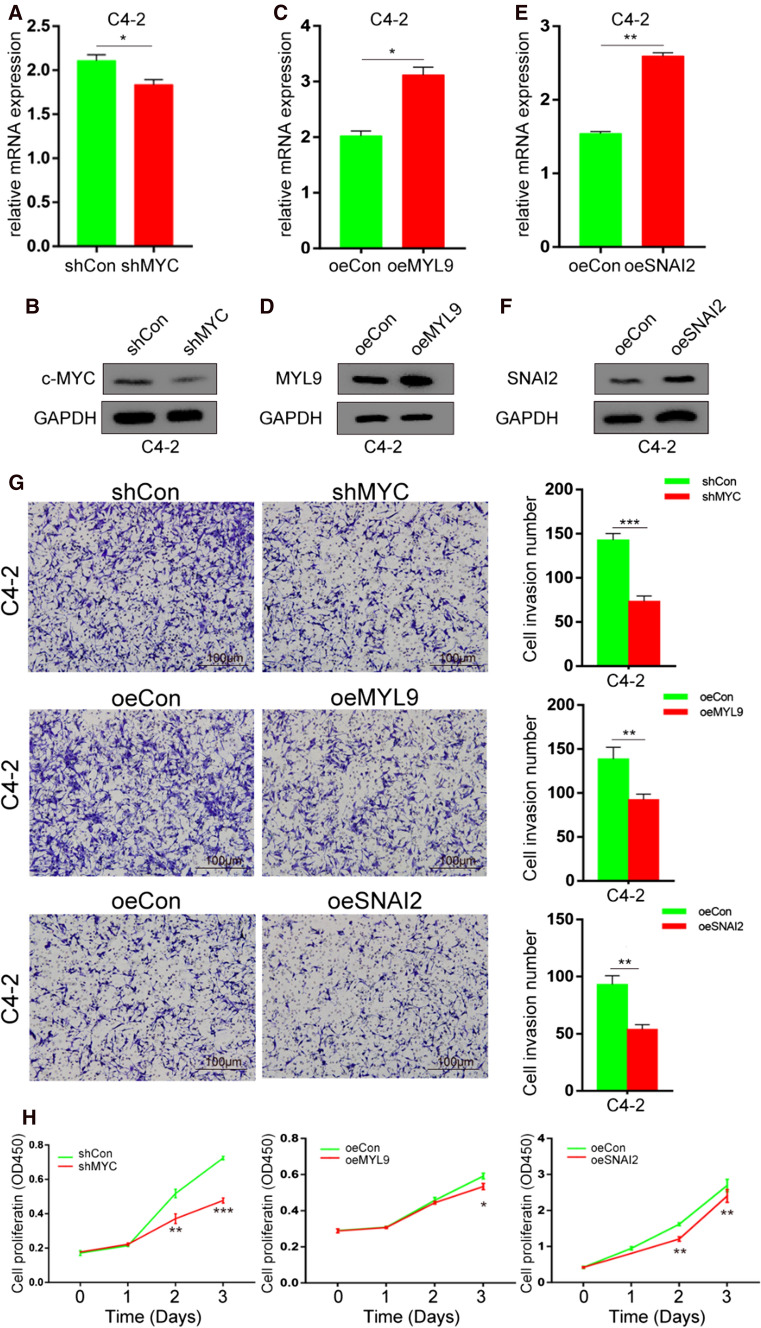
MYC, MYL9 and SNAI2 influence C4-2 cell invasion and proliferation. **A**, **B** The mRNA and protein expression of MYC after MYC knock-down (shMYC) plasmids transfected into C4-2 PCa cells. **C**, **D** The MYL9 mRNA and protein level after overexpression MYL9 (oeMYL9) plasmids transfected into C4-2 cells. **E**, **F** The expression of SNAI2 in both mRNA and protein level after C4-2 cells transfected by overexpression SNAI2 (oeSNAI2) plasmids. **G** The invasion ability of C4-2 cells after transfection with different plasmids. **H** The proliferative ability of C4-2 cells after transfection with different plasmids. The expression level of mRNA and protein was used GAPDH as inner control. * represents *P* < 0.05, ** represent *P* < 0.01, *** represent *P* < 0.001

To further verify the function of the three hub genes in PCa cell, we constructed *MYC* knockdown (shMYC) plasmids, *MYL9* overexpression (oeMYL9) and *SNAI2* overexpression (oeSNAI2) plasmids. We found that we successfully knock-down *MYC* in C4-2 cells (Fig. 10A, B). The oeMYL9 and oeSNAI2 C4-2 cells were constructed successfully, too (Fig. 10C–F). Then, we detected cells invasion and proliferation ability after C4-2 cell transfected by different plasmids. We found that after transfected by shMYC, oeMYL9 and oeSNAI2 plasmids, the C4-2 cells invasion ability will decrease (Fig. 10G). When C4-2 cells transfected with shMYC, oeMYL9 and oeSNAI2 plasmids, the cell proliferation will decrease, too (Fig. 10H).

## Discussion

PCa and BPH are common encountered diseases in men older than 70 [23]. In recent decades, because of changes in the global population structure and aging, elderly males have increased in number [24, 25]. So, these two diseases seriously threaten the health of elder men. Many studies had reported that there is a correlation between BPH and PCa. However, the genetic relationship between these two diseases is still unclear. Understanding the gene mutation between BPH and PCa can help finding potential biomarkers for predicting the occurrence of PCa from BPH.

Over recent decades, bioinformatics on microarray data have focused broadly on the PCa occurrence depend on bioinformatic analysis and have revealed some of the mechanisms that may lead to PCa. Trying to find the genetic relationship between BPH and PCa, we made a bioinformatic study. A total of 60 DEGs were found from GEO database. In addition, DEGs were mainly enriched in pathways associated with in proteoglycans in cancer, inositol 1, 4, 5 trisphosphate binding, and negative regulation of nitric oxide biosynthetic process which has been reported important in the occurrence of cancers [26–28]. Then, depending PPI network, we found 7 hub genes among 60 DEGs.

Hub genes, *MYC*, *CXCR4*, *CSRP1*, *SNAI2*, *MYL9*, *ACTG2*, and *MYH11*, were found. This result indicates that mutations in these genes may play significant roles in the development of PCa from BPH. These genes function in cancer have been widely reported. MYC (MYC proto-oncogene) affects PCa progression due to a high-fat diet and plays a positive role in regulating the androgen receptor and androgen-receptor splice variants in PCa [29, 30]. CXCR4 (C-X-C motif chemokine receptor 4) may promote PCa metastasis through regulation of phosphatidylinositol 4-kinase IIIα (PI4KIIIα) and SAC1 phosphatase [31]. In addition, CXCL12/CXCR4 can increase the malignancy of breast cancer and cervical cancer [32, 33]. SNAI2 (snail family transcriptional repressor 2) can regulate prostate tumor progress, angiogenesis, and metastasis potentially by modulating the GSK-3β/β-catenin signaling pathway [34]. SNAI2 also participates in regulation of the initiation and metastasis of breast cancer cells [35]. MYL9 (myosin light chain 9) can predict malignant progression and poor biochemical recurrence-free survival of PCa [36]. MYL9 is also associated with the recurrence of colorectal cancer [37]. ACTG2 (actin gamma 2, smooth muscle) and MYH11 (myosin heavy chain 11) have an affection in the development of PCa [38, 39]. ACTG2 can also affect hepatocellular carcinoma cell migration and tumor metastasis, and MYH11 may play a pivotal function in the progression of lung cancer and bladder cancer [40, 41].

The above results have proven that these 7 hub genes can be markers for predicting the occurrence of PCa from BPH. Then, trying to understand the role of these 7 hub genes in PCa progression, we analyzed the expression of these 7 hub genes in different databases including TCGA and CPGEA. When analyzing hub gene expression depending on TCGA data, we found 2 up-regulated hub genes, *MYC* and *CXCR4*, were not expressed differently between normal prostate samples and PCa samples in the GEPIA database. These results may due to that the normal sequence from GEPIA includes data from both TCGA and GTEx database. So, we detected these hub genes expression in different databases, we found that all these hub genes except *CXCR4* can lead to the occurrence of PCa, but not in Chinese population. In addition, some hub genes expression differently in different tumor stage. That means some hub genes can be indicators to predict disease progression. In addition, we also found that *CSRP1*, and *MYH11* can influence patients’ DFS. These results reflect that these genes can not only serve as markers for predicting PCa from BPH but can also reflect the severity of PCa.

Finally, we utilized clinical specimens and C4-2 PCa cells to validate the hub genes’ functions in promoting PCa development. We found that *MYC* was higher expression and *MYL9*, and *SNAI2*, were lower expression when BPH patients suffered PCa. These results indicated that these three genes can be biomarkers for predicting the occurrence of PCa from BPH. Then, we transfected different plasmids into C4-2 PCa cells. We found that *MYC* can increase cell invasion and proliferation ability. In addition, *MYL9* and *SNAI2* can decrease cell invasion and proliferation ability. The results confirmed that the three hub genes can influence the progression of PCa.

However, our study had some limitations. First, although we included 3 datasets in the study, the number of samples was still small; there were only 10 BPH samples and 24 PCa samples. In addition, the number of clinical samples was small, too. A too-small sample size may have led to a less representative study. Second, although we validated the hub genes’ expression in different databases and clinical samples, the mechanisms of these hub genes in leading to PCa from BPH did not study. Thus, our results require further validation. Finally, we analyzed the clinical value of these hub genes in different databases to make our results more comprehensive. But this process was depended on the data from normal prostate tissues and PCa tissues. This may lead to bias. So, in the future, more studies need to make to prove the results we found and to find the mechanism of how BPH can develop into PCa. But, we made the first study to find potential markers to predict PCa from BPH and analyzed their clinical value in PCa.

## Conclusion

We used bioinformatic analysis to identify seven significant genes may be potential biomarkers for predicting the occurrence of PCa from BPH. In addition, these hub genes can also affect the progression of PCa.

## Supplementary Information

Below is the link to the electronic supplementary material.

Supplementary file 1 (TIF 957 KB)—Figure S1. (A) The residuals plot of logistic regression. (B) The normal P–P plot of standardized residuals of logistic regression.

Supplementary file 2 (TIF 3646 KB)—Figure S2. The OS of 7 hub genes in PCa patients was evaluated by Kaplan-Meier curve from GEPIA. (A) *MYC* (B) *CXCR4* (C) *CSRP1* (D) *SNAI2* (E) *MYL9* (F) *ACTG2* (G) *MYH11*.

Supplementary file 3 (TIF 6675 KB)—Figure S3. The expression of 7 hub genes in different databases on TCGA data. (A) The expression of 7 hub genes in PCa depend on UALCAN database. (B) The expression of 6 hub genes in PCa depend on The Human Protein Atlas.

Supplementary file 4 (DOCX 5164 KB)

Supplementary file 5 (DOC 95 KB)

Supplementary file 6 (DOCX 17 KB)

## Data Availability

The RNA-sequence and clinical data can get from different public databases including GEO (https://www.ncbi.nlm.nih.gov/gds), TCGA (https://www.cancer.gov/about-nci/organization/ccg/research/structural-genomics/tcga), and CPGEA (https://www.cpgea.com). Other data can get from the corresponding author.
