# Supplementary figures and images for: Identification of hub genes predicting the development of prostate cancer from benign prostate hyperplasia and analyzing their clinical value in prostate cancer by bioinformatic analysis
Source: Discov Oncol. 2022 Jun 30;13:54. doi: 10.1007/s12672-022-00508-y (PMC9243208; doi:10.1007/s12672-022-00508-y)

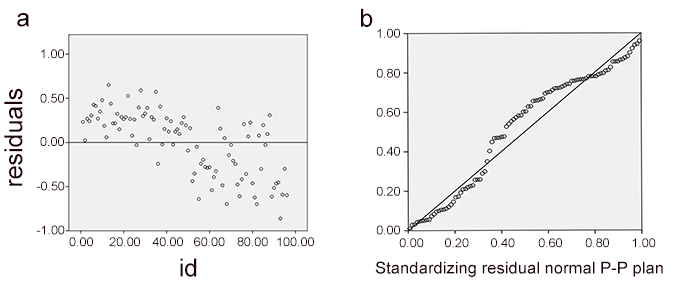

Supplement: Supplementary file 1 — (TIF 957 KB)—Figure S1. (A) The residuals plot of logistic regression. (B) The normal P–P plot of standardized residuals of logistic regression. [file 12672_2022_508_MOESM1_ESM.tif]

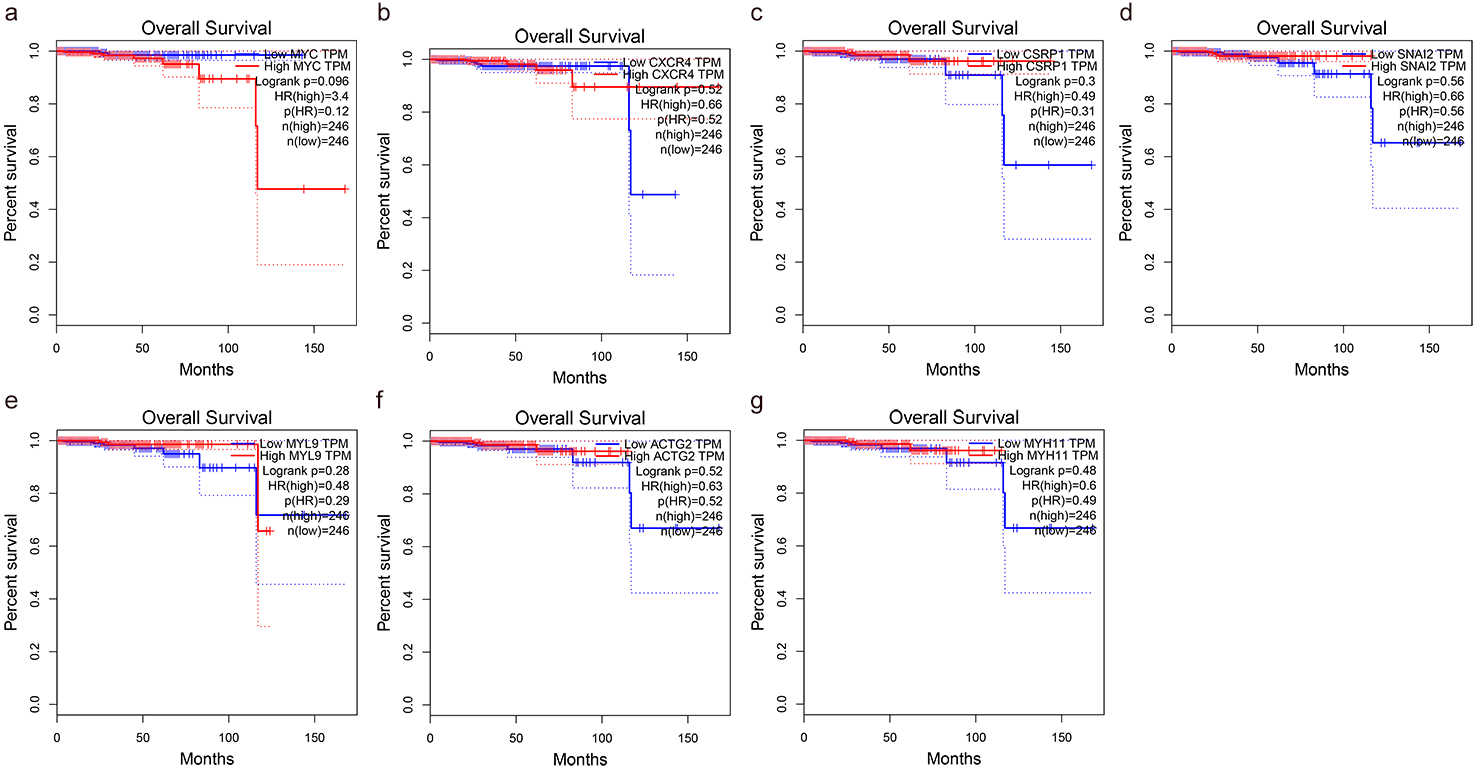

Supplement: Supplementary file 2 — (TIF 3646 KB)—Figure S2. The OS of 7 hub genes in PCa patients was evaluated by Kaplan-Meier curve from GEPIA. (A) MYC (B) CXCR4 (C) CSRP1 (D) SNAI2 (E) MYL9 (F) ACTG2 (G) MYH11. [file 12672_2022_508_MOESM2_ESM.tif]

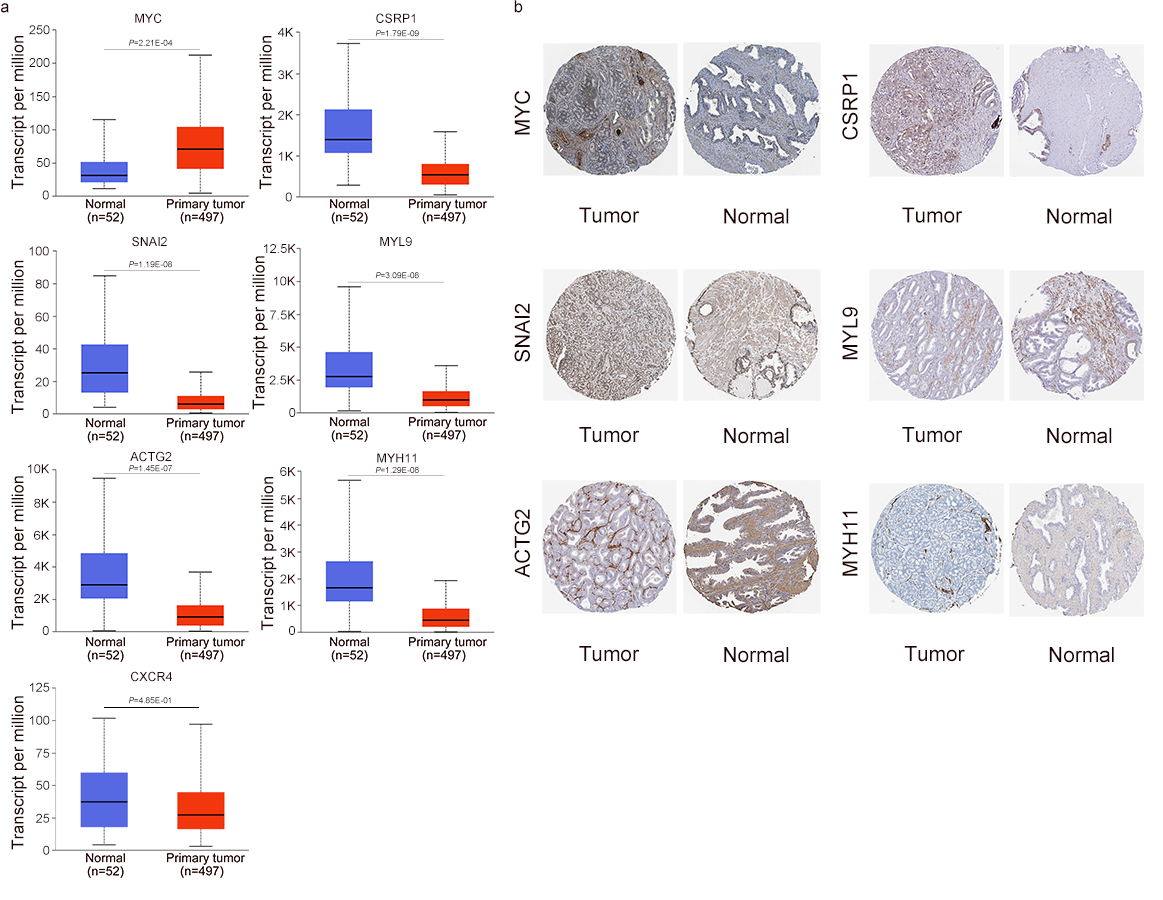

Supplement: Supplementary file 3 — (TIF 6675 KB)—Figure S3. The expression of 7 hub genes in different databases on TCGA data. (A) The expression of 7 hub genes in PCa depend on UALCAN database. (B) The expression of 6 hub genes in PCa depend on The Human Protein Atlas. [file 12672_2022_508_MOESM3_ESM.tif]

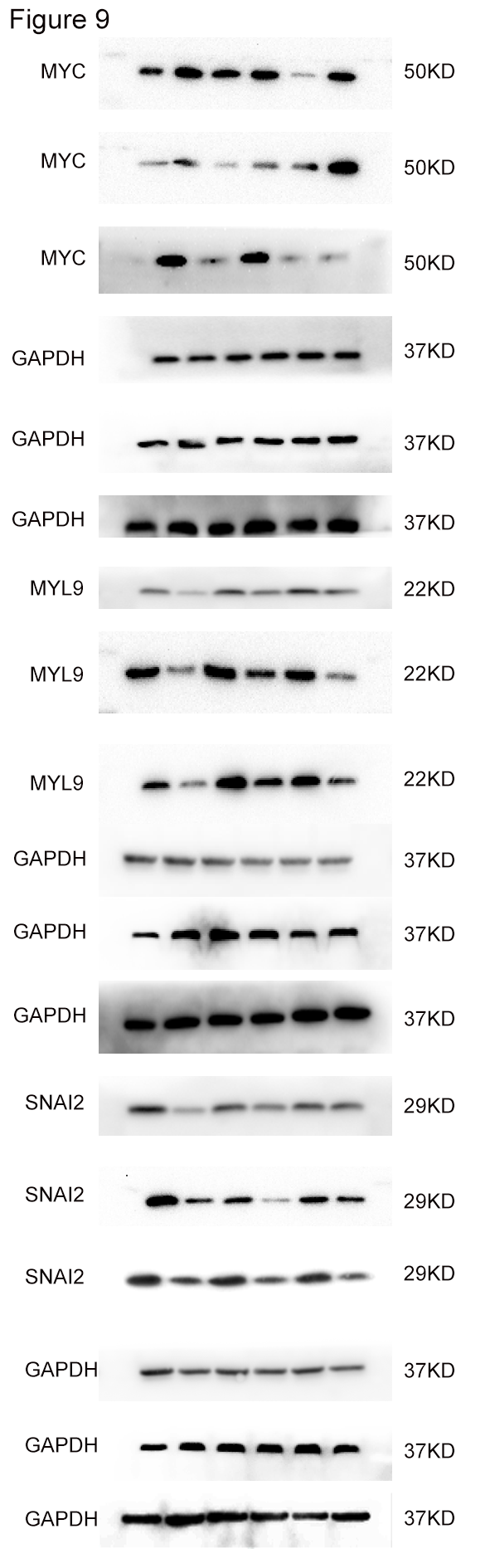


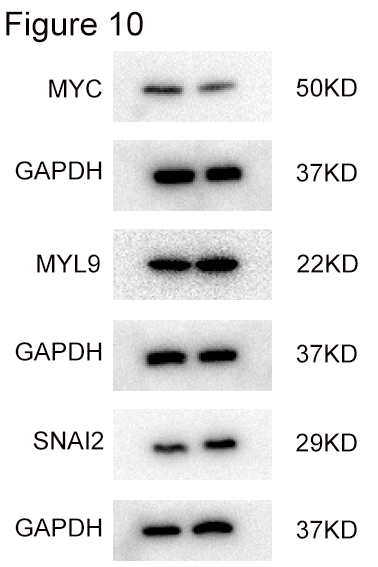

Supplement: Supplementary file 4 — (DOCX 5164 KB) [file 12672_2022_508_MOESM4_ESM.docx]
