# Supplementary material for: Identification of hub genes predicting the development of prostate cancer from benign prostate hyperplasia and analyzing their clinical value in prostate cancer by bioinformatic analysis
Source: Discov Oncol. 2022 Jun 30;13:54. doi: 10.1007/s12672-022-00508-y (PMC9243208; doi:10.1007/s12672-022-00508-y)
Supplement: Supplementary file 5 — (DOC 95 KB) [file 12672_2022_508_MOESM5_ESM.doc]

Table S1: The characteristic of sixty DEGs from three GSE datasets

| Gene name | Expression trend | *P*-value(GSE5377) | *P*-value(GSE104749) | *P*-value(GSE30994) |
| --- | --- | --- | --- | --- |
| UAP1 | high | 3.08E-04 | 2.14E-03 | 1.60E-03 |
| PCP4 | high | 1.24E-02 | 3.69E-02 | 4.34E-03 |
| TRIB1 | high | 2.52E-05 | 3.22E-05 | 9.51E-05 |
| PDLIM5 | high | 7.48E-03 | 2.73E-02 | 1.02E-02 |
| DKK3 | high | 5.42E-04 | 1.39E-02 | 2.31E-02 |
| PRR16 | high | 4.77E-02 | 1.02E-03 | 1.61E-05 |
| MEIS2 | high | 4.63E-02 | 4.01E-02 | 3.95E-02 |
| BAMBI | high | 3.80E-02 | 3.13E-02 | 8.22E-05 |
| MCCC2 | high | 1.38E-02 | 5.01E-03 | 5.90E-04 |
| CACNA1D | high | 1.32E-02 | 6.63E-03 | 2.43E-02 |
| CAV1 | high | 1.64E-06 | 1.47E-06 | 2.98E-02 |
| DAPK1 | high | 3.60E-02 | 2.00E-03 | 4.21E-02 |
| MYC | high | 2.67E-03 | 8.66E-04 | 9.13E-03 |
| SIM2 | high | 1.01E-02 | 1.81E-03 | 3.31E-02 |
| CXCR4 | high | 8.82E-03 | 1.91E-02 | 1.51E-02 |
| MYO6 | low | 5.74E-03 | 3.62E-02 | 3.29E-02 |
| RGN | low | 6.06E-05 | 1.76E-02 | 1.18E-02 |
| HEPH | low | 3.98E-02 | 8.87E-04 | 8.07E-03 |
| MAOA | low | 1.52E-07 | 1.39E-02 | 1.14E-03 |
| NCAM1 | low | 1.93E-05 | 3.13E-02 | 1.49E-03 |
| KRT15 | low | 6.68E-04 | 3.09E-03 | 1.10E-03 |
| FHL1 | low | 3.04E-02 | 2.38E-02 | 3.79E-04 |
| TACC1 | low | 1.22E-05 | 8.50E-03 | 3.06E-02 |
| AGL | low | 4.03E-06 | 1.21E-03 | 2.49E-04 |
| ATP2B4 | low | 8.59E-03 | 1.04E-02 | 8.71E-03 |
| MYL9 | low | 1.53E-05 | 2.03E-02 | 1.41E-04 |
| CYP4B1 | low | 2.18E-03 | 2.37E-03 | 1.05E-02 |
| HSPB8 | low | 1.14E-03 | 2.31E-04 | 5.61E-05 |
| SPOCK3 | low | 3.29E-03 | 4.05E-02 | 7.54E-06 |
| LGALS3BP | low | 1.08E-02 | 8.98E-03 | 1.03E-02 |
| MYH11 | low | 6.04E-04 | 2.81E-02 | 1.40E-05 |
| ITPR1 | low | 1.45E-02 | 1.20E-04 | 1.91E-04 |
| TNS1 | low | 3.02E-02 | 1.40E-03 | 4.02E-05 |
| PER3 | low | 1.70E-02 | 1.76E-02 | 1.30E-04 |
| DPT | low | 4.46E-02 | 5.59E-04 | 7.43E-03 |
| KRT14 | low | 1.32E-04 | 1.70E-03 | 4.25E-05 |
| GSTM3 | low | 3.67E-02 | 3.13E-03 | 3.45E-04 |
| ZNF185 | low | 1.09E-02 | 2.37E-03 | 7.63E-06 |
| GPRC5B | low | 1.60E-04 | 4.31E-02 | 2.76E-03 |
| WNT5B | low | 2.92E-04 | 1.75E-02 | 1.78E-03 |
| GPC1 | low | 6.33E-03 | 5.70E-03 | 5.41E-04 |
| PTGIS | low | 1.31E-04 | 5.72E-03 | 1.78E-04 |
| BDH2 | low | 9.41E-03 | 4.01E-02 | 2.17E-03 |
| LAMB3 | low | 2.41E-02 | 5.50E-03 | 5.03E-04 |
| CSRP1 | low | 5.79E-06 | 2.47E-02 | 5.12E-04 |
| KCNAB1 | low | 2.94E-02 | 2.12E-02 | 8.07E-04 |
| MYLK | low | 7.58E-04 | 4.26E-02 | 8.90E-04 |
| TGFBR3 | low | 6.26E-04 | 1.48E-02 | 6.62E-04 |
| RPRM | low | 4.58E-04 | 3.36E-03 | 1.67E-02 |
| TRIM36 | low | 1.14E-03 | 2.21E-02 | 3.11E-03 |
| FLNC | low | 3.33E-04 | 3.89E-03 | 7.30E-04 |
| AOC3 | low | 1.91E-03 | 1.83E-02 | 5.20E-05 |
| ASPA | low | 6.86E-04 | 2.39E-03 | 9.64E-05 |
| ATP1A2 | low | 1.23E-05 | 1.56E-03 | 4.22E-04 |
| PLCL1 | low | 3.03E-04 | 2.97E-03 | 3.04E-03 |
| CADM1 | low | 2.86E-04 | 3.46E-03 | 1.71E-02 |
| ACTG2 | low | 3.63E-03 | 1.53E-02 | 6.85E-03 |
| PPP1R3C | low | 4.66E-03 | 3.20E-02 | 5.88E-04 |
| SNAI2 | low | 1.19E-04 | 3.95E-03 | 1.26E-03 |
| PRKCA | low | 6.42E-03 | 3.64E-02 | 1.30E-02 |
