# Supplementary material for: Identification of hub genes predicting the development of prostate cancer from benign prostate hyperplasia and analyzing their clinical value in prostate cancer by bioinformatic analysis
Source: Discov Oncol. 2022 Jun 30;13:54. doi: 10.1007/s12672-022-00508-y (PMC9243208; doi:10.1007/s12672-022-00508-y)
Supplement: Supplementary file 6 — (DOCX 17 KB) [file 12672_2022_508_MOESM6_ESM.docx]

Table S2: The characteristics of patients included in the study

| Name | Age | Age in diagnosis | TNM | Gleason Score |
| --- | --- | --- | --- | --- |
| Luo ✳✳ | 77 | 72 | T2N1M0 | 3+4 |
| Tang ✳✳ | 75 | 72 | T2N0M0 | 3+4 |
| Zhang ✳✳ | 82 | 76 | T3N0M0 | 4+4 |
| Wu ✳✳ | 73 | 68 | T3N1M0 | 4+4 |
| Wang ✳✳ | 67 | 66 | T4N1M1 | 4+5 |
| Ling ✳ | 84 | 80 | T2N0M0 | 3+4 |
| Liu ✳✳ | 82 | 78 | T2N0M0 | 3+4 |
| Zhang ✳✳ | 72 | 69 | T3N1M0 | 5+4 |
| Jin ✳✳ | 71 | 69 | T4N0M0 | 4+4 |
| Gu ✳✳ | 67 | 62 | T2N0M0 | 3+4 |
| Shen ✳✳ | 74 | 68 | T3N0M0 | 3+4 |
| Shi ✳✳ | 89 | 85 | T3N1M0 | 4+4 |
| Meng ✳✳ | 67 | 65 | T4N1M1 | 5+4 |
| Zhao ✳✳ | 84 | 82 | T3N0M0 | 4+3 |
| Pan ✳✳ | 81 | 74 | T2N0M0 | 4+3 |
| Zhao ✳✳ | 75 | 69 | T3N0M0 | 4+4 |
| Feng ✳ | 83 | 89 | T2N0M0 | 3+4 |
| Chen ✳✳ | 76 | 73 | T3N0M0 | 4+4 |
